# Effect of Cement Substitution with Mineral Fillers on NO_x_ Air-Purification Efficiency and Photocatalytic Reaction Selectivity of Nano-TiO_2_-Modified Cementitious Composites

**DOI:** 10.3390/ma17235775

**Published:** 2024-11-25

**Authors:** Karol Chilmon, Maciej Kalinowski, Wioletta Jackiewicz-Rek

**Affiliations:** Faculty of Civil Engineering, Warsaw University of Technology, 00-637 Warsaw, Poland; maciej.kalinowski@pw.edu.pl (M.K.); wioletta.rek@pw.edu.pl (W.J.-R.)

**Keywords:** cement, concrete, photocatalysis, air purification, nitrogen oxides, titanium dioxide, nanomaterials

## Abstract

This research investigated the properties of photocatalytic cementitious composites, including their air-purification efficiency. A method of characterizing the removal of airborne pollutants (nitrogen oxides), simulating the actual NO_x_ concentration and irradiation conditions in Warsaw, Poland, in the autumn/winter season was established. The study analyzed the impact of changes in the composition of cement mortars—partial substitution of the binder with mineral fillers—on the properties of the external photoactive surface of the composite. The designed experimental plan included both quantitative and qualitative variables (type and amount of fillers used). It was found that the photocatalytic performance of the composite was correlated with its pore total content and pore size distribution—the higher the content of mineral fillers, the lower the porosity and the less effective its photocatalytic properties. The selectivity of the photocatalytic NO_x_ reactions also deteriorated as the content of the mineral fillers increased. The study confirmed the validity of increasing the binder content in cementitious composites to enhance their photocatalytic performance.

## 1. Introduction

Air pollution represents one of the vital global challenges with substantial implications for public health, environmental stability, and climate [1,2]. Key pollutants like nitrogen oxides (NO_x_), sulfur oxides (SO_x_), volatile organic compounds (VOCs), carbon monoxide (CO), and particulate matter (PM) are predominantly produced by anthropogenic sources—industrial activities, transportation, and energy production [3]. These pollutants contribute to serious health issues, including respiratory and cardiovascular diseases [1]. The World Health Organization (WHO) estimates that millions of premature deaths worldwide are attributable to exposure to air pollution, emphasizing the urgent need for practical, sustainable solutions to reduce atmospheric contaminants, especially within urbanized and industrial regions [4].

In light of this challenge, photocatalytic materials have gained attention as a potential solution for passive air purification [5,6,7]. Unlike conventional methods that rely on filtration or capture mechanisms, photocatalytic materials degrade pollutants through light-induced chemical reactions. Due to its chemical stability and cost-effectiveness, titanium dioxide (TiO_2_) is the most widely studied and applied photocatalyst. When exposed to electromagnetic radiation of specific wavelength and atmospheric gases, TiO_2_ undergoes a photocatalytic reaction that generates hydroxyl radicals (OH^·^) and superoxide anions (O_2_^·−^). These radicals participate in oxidation reactions of gaseous air pollutants, converting them to their ionic forms (e.g., NO_3_^−^ in the case of NO_x_) [8] or to CO_2_ and H_2_O (for polycyclic aromatic hydrocarbons like benzene) [9]. Free radicals can also penetrate bacterial cells, leading to their degradation (self-disinfection effect) [10], and degrade contaminant particles (self-cleaning effect) [11].

Photocatalytic materials can be incorporated into cementitious composites, paving the way for large-scale applications in urban infrastructure. This approach has gained traction, especially in cities where concrete surfaces—pavements, roads, and building facades—are prevalent [12,13,14]. As a passive air-purifying technology, photocatalytic cementitious composites (PCCs) offer a low-maintenance, sustainable solution for urban air quality improvement, potentially over large areas, without the need for any energy source other than sunlight to operate.

Despite the potential of PCCs, optimizing their performance in real-world applications remains a challenge. Their efficiency depends on several factors, including the type of pollutant, light intensity, environmental conditions, and composite composition. Among the environmental factors, the most significant are the irradiance of the radiation [15,16] (particularly in the UV-A and UV-B bands), relative air humidity [17,18], air flow rate [19], and surface cleanliness [20]. From the material perspective, the porosity of the structure in the near-surface zone [7] (and the factors that determine it), the content of photocatalyst in the composite mass [13], and its specific surface area and uniformity of distribution in the cement matrix, as well as the characteristics of the composite’s surface itself influence the overall performance of the photoactive external layer of the composite that is exposed to the elements [16,21,22]. Among the technological factors, the parameters of the forming process regarding the uniformity of distribution of the photocatalyst particles and their highest possible concentration on the composite surface, as well as the course of the curing process that shapes the porosity of the structure in the near-surface zone, have a statistically significant influence over the photocatalytic performance of the composite [8].

Each of the aforementioned factors can significantly impact the photocatalytic properties of cement composites, potentially rendering them almost non-existent if not meticulously considered during the design and application. In cement-based composites, the reactions of the constituents of the cement matrix primarily depend on the composite’s surface interactions with environmental factors [23]. These surfaces are exposed to fluctuating parameters, including temperature, humidity, and exposure to pollutants, all of which evolve as the composite experiences mechanical and environmental stress—the only aspect that can be fully controlled during the design process is the influence of the composite’s composition on its photocatalytic performance.

Mineral fillers are of growing interest to the cement industry, especially as partial substitutes for Portland cement [24,25]. Their use is prompted by their dual benefits of reducing the carbon footprint associated with cement production and optimizing material properties. However, the addition of mineral fillers could impact the photocatalytic properties of cement composites in both beneficial and detrimental ways, depending on the type and amount of filler used. Photocatalytic reactions are typically facilitated by the presence of titanium dioxide (TiO_2_), which is activated by UV light to break down pollutants like nitrogen oxides (NO_x_) on the composite surface [26,27]. As mineral fillers are introduced, their physical and chemical interactions with TiO_2_ and other cementitious materials can alter the efficiency of these photocatalytic reactions. For instance, quartz and chalcedonite powders have specific surface properties and particle sizes that could affect the distribution and surface exposure of TiO_2_ within the composite. A homogeneous dispersion of the photocatalyst is essential to maintaining active sites on the composite’s surface; an uneven distribution can reduce the number of exposed photocatalytic sites, thereby decreasing the material’s pollutant decomposition capability.

Moreover, mineral fillers influence the porosity and microstructure of the cement matrix, which are critical factors for the adsorption and reaction rates of pollutants on the composite surface—fillers that densify the composite’s structure may reduce porosity, limiting access to the photocatalyst and thus decreasing the material’s photocatalytic efficiency. In this context, the authors aimed to investigate how a partial replacement of binder with mineral fillers would affect the cement composite’s ability to decompose nitrogen oxides (NO_x_)—a key measure of photocatalytic performance.

## 2. Research Framework

A two-factor experimental plan was designed, using the type of mineral powder (qualitative variable) and the degree of binder replacement (quantitative variable) as the independent variables. In total, five mortar series were prepared (Table 1), all of which were designed as self-leveling mortars.

**Figure 1:**
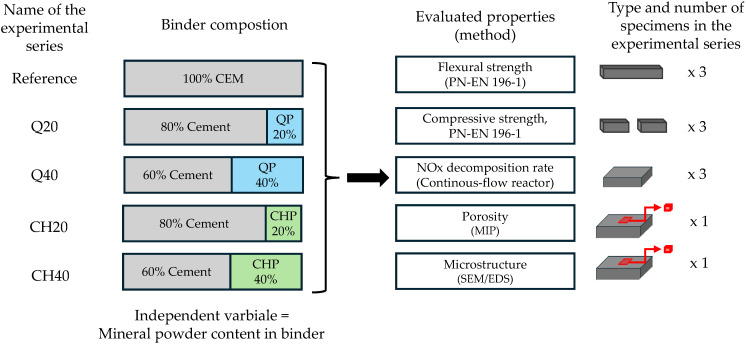
The experimental framework (abbreviations: QP—quartz powder; CHP—chalcedonite powder).

All composites were characterized by constant mass coefficients—w/b (water-to-binder ratio), b/s (binder-to-sand ratio), and constant photocatalyst content. The content of the PCE superplasticizer varied between series, as the consistency of the mortars was set in the range of 280–320 mm, which was measured using the free-flow method. The dependent variables in the research plan were tensile and compressive strength, pore network characteristics, and the efficiency and selectivity of photocatalytic decomposition of nitrogen oxides (Figure 1).

The microstructure of the mortars was also assessed regarding the uniformity of distribution of photocatalyst particles on the photoactive external surface. The properties of the Portlandite cement, powders, and sands were evaluated as described in Section 3.

## 3. Materials and Methods

### 3.1. Materials

CEM I 42.5 R cement (compliant with the PN-EN 197-1 [28] standard; Ożarów, Poland) was used to prepare the cement mortars. Its properties were confirmed regarding its mechanical performance (according to PN-EN 196-1 [29]), initial and final setting time (acc. To PN-EN 196-3 [30]), specific gravity, and specific surface area (using Blain method) (Table 2).

**Figure 2:**
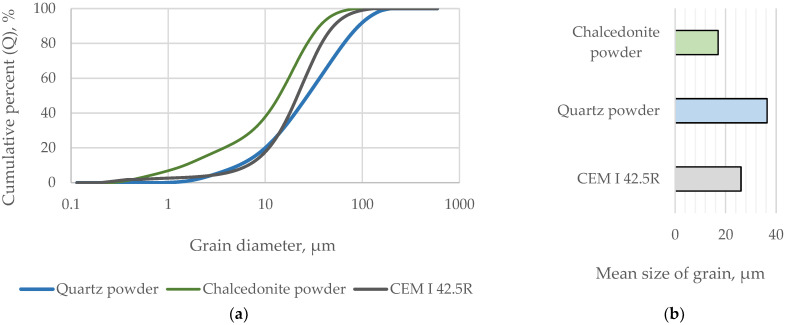
(**a**) Grain size distribution. (**b**) Average grain diameter of cement and mineral fillers used in the study.

The investigated mineral fillers were characterized in terms of chemical composition and grain size distribution (presented in Table 3 and Figure 2).

Both fillers had similar chemical compositions and densities, which resulted from similarities in the mineralogical composition of the rocks from which they were obtained. The composition of both fillers was dominated by silicon compounds (>90%) (quartz in the case of quartz powder, and chalcedony in the case of chalcedonite powder). The presence of trace amounts of aluminum and iron compounds was also noted, which was due to the heterogeneity of the deposit. Both powders were mechanically ground and found to have an irregular/angular grain morphology. The chalcedonite powder was characterized by much higher fineness than the quartz filler—the average grain size was 17.0 μm for chalcedonite powder, 26.1 μm for Portland cement, and 39.3 μm for quartz powder (Figure 2).

A mix of two photocatalytic materials was used in the study (first and second generation): TiO_2_ (A) (K7000; Leverkusen, Germany) and TiO_2_ (B) (P25; Shanghai, PRC), whose properties in the powder state are described in [31]. The content of the individual crystalline phases, the size of the crystallites, and the specific surface area of the used modifiers are presented in Table 4. The mass ratio between the two photocatalysts, as well as the total TiO_2_ content, was kept at the same level in each of the investigated cement mortars (the TiO_2_ (A)-to-TiO_2_ (B) ratio was 0.25 [-], and the total TiO_2_ content was 12.5 kg/m^3^). A mix of two photocatalytic materials was used to expand the scope of the light spectrum in which the composite would exhibit photocatalytic performance—first-generation TiO_2_ (P25) is primarily active in the UV-a spectrum while the second-generation (K7000) activation band expands into the visible light spectrum.

**Figure 3:**
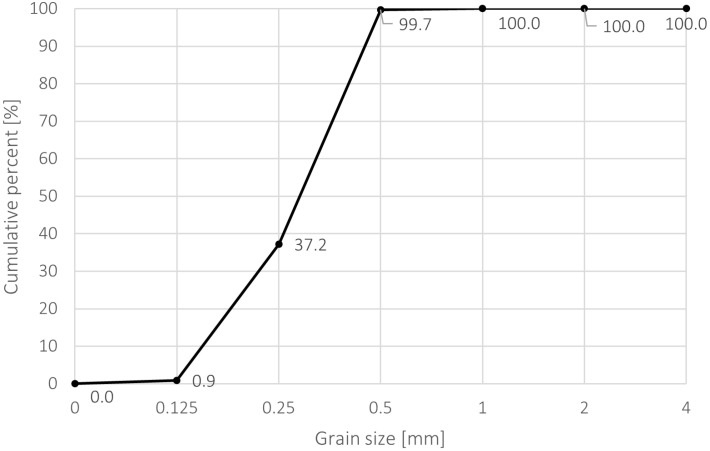
Grain size distribution of sand used in the study.

This study used fire-dried quartz sand aggregate with a granulation of 0.1/0.5 (Corrado, Poland), which meets the requirements of EN 13139 [32] (Table 5 and Figure 3).

### 3.2. Methods

The tensile and compressive strength of the investigated mortars was determined after two days of curing following the PN-EN 196-1 protocol using three prism-shaped specimens with dimensions of 40 × 40 × 160 mm [29]. The mortar samples were de-molded 24 h after preparation and then stored in water until further tests were performed (temp = 20 ± 2 °C).

The porosity of the hardened mortars was determined by mercury intrusion porosimetry (Autopore IV 9510, Micrometritics, Norcross, GA, USA). The tests used samples measuring 10 × 10 × 10 mm, which were mechanically cut from 40 × 40 × 160 mm samples. The uncut samples were stored in water for 28 days, and then mechanically cut and dried at 105 °C for 48 h. The measurement range included pores with sizes from about 0.003 to 360 μm. The equivalent pore radius was determined from the Washburn equation (Equation (1)), where *r* is the pore radius [m], *γ* is the surface tension of the liquid [N/m], θ is the contact angle between the liquid and the solid surface [rad], and *P* is the applied pressure or capillary pressure [Pa].

(1) $$r=\frac{-2\gamma \cos\theta }{P}$$

The effectiveness in gaseous pollutant (NO_x_) removal from air by the photocatalytic cementitious composites was calculated using a test procedure developed by the authors based on ISO 22197 [33]. Three cuboid samples with dimensions of 40 × 140 × 160 mm were prepared for each investigated mortar series. After demolding, the samples were cured for 28 days in a curing chamber (RH > 95%, temp = 20 ± 2 °C). The samples made for the air-purification tests were not cured in water to prevent phenomena associated with an uncontrolled change in the surface characteristics of the composites from occurring (efflorescence, precipitates of unknown origin, etc.) that could influence the photocatalytic performance of the photoactive external surface of the investigated mortars and reduce the quality of the experiment.

Before the air-purification tests, the samples underwent a preparation phase in which the external photoactive surface was cleaned of contaminants. Firstly, the surface was washed with distilled water and scrubbed. Afterward, the samples were dried at 60 °C for two hours and placed in an irradiation chamber for 16 h, where, under 10 W/m^2^ UV-a irradiation, organic impurities on the photoactive surface were burned away. In the last step, the samples were again rinsed with distilled water and dried at 60 °C. The efficiency of NO_x_ removal from air was measured 2 h after the end of the sample preparation at the earliest.

**Figure 4:**
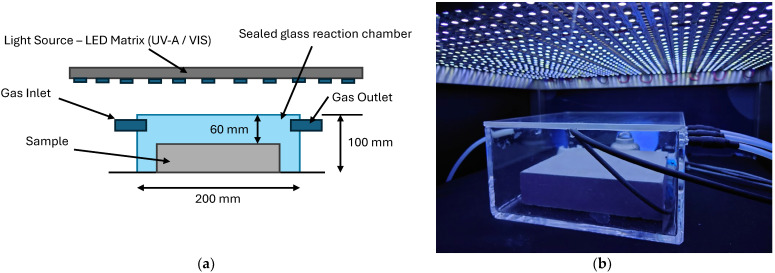
(**a**) Diagram of the setup for testing the NO_x_ decomposition efficiency. (**b**) A reaction chamber containing a sample inside.

For the test, the prepared cementitious samples were placed in a sealed glass reaction chamber (4 dm^3^ in volume) with an investigated photoactive surface facing the light source (Figure 4). The gas flow in the reactor during the test was 2 L/min. The nitrogen oxide concentration at the chamber entrance was 100 ± 5 ppb and was maintained throughout the test using a multi-gas calibrator. The temperature and relative humidity were kept constant throughout the test (RH = 40 ± 5%, temp = 20 ± 2 °C). The test was performed in an air environment.

Two low-radiation LED lighting sources were used in the tests, reproducing the actual lighting conditions encountered in Poland during autumn/winter—UV-A (ultraviolet light): 365 nm and 1.0 W/m^2^; and VIS (visible light): global radiation of 150 W/m^2^. The VIS radiation source did not emit radiation below 400 nm. The concentration of NO and NO_x_ during the test was recorded using a Teledyne API T200 chemiluminescence detection analyzer (San Diego, CA, USA).

**Figure 5:**
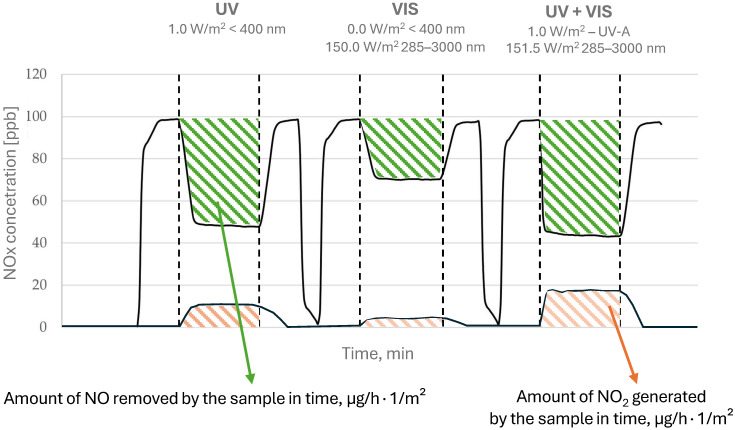
A schematic of the test procedure for NO_x_ removal from air under different irradiation conditions.

The study was divided into several stages (Figure 5). The NO_x_ concentration in the reactor was maintained at a constant level of 100 ± 5 ppb. Subsequently, the selected light sources were alternately turned on and off during the test to establish three distinct irradiation conditions: visible light only, UV-A light only, and combined visible and UV-A light.

Each irradiation phase lasted 40 min, with 30 min intervals without any active light source between phases, which had one or more active sources. The amount of NO introduced into the reactor, NO removed by the photocatalytic sample, and the NO_2_ produced during the photocatalytic process for each radiation phase were calculated. Based on the data, the removal/generation rates, expressed as the absolute reduction/generation of NO_x_ in micrograms per hour per square meter of photocatalytic cement matrix (µg/h × 1/m^2^), under various irradiation conditions were calculated. The selectivity of NO_x_ removal was calculated using Equation (2) based on the NO/NO_2_ amounts determined from the areas highlighted in Figure 5.

(2) $$S=1-\frac{{generated\;NO}_{2}}{removed\;NO}\;[\text{-}]$$

The distribution of the photocatalysts on the mortar sample surfaces was examined using Scanning Electron Microscopy coupled with energy-dispersive X-ray spectroscopy (SEM/EDS). The analyses were conducted on approximately 20 × 20 × 10 mm mortar samples, sectioned from the specimens’ central region, which initially measured 40 × 40 × 160 mm. The EDS titanium distribution maps underwent graphic processing to exclude weak signal points that potentially originated from titanium within the binder or aggregate matrix. The processed images were binarized, and the pixel count corresponding to the titanium signal was quantified using histogram analysis.

**Figure 6:**
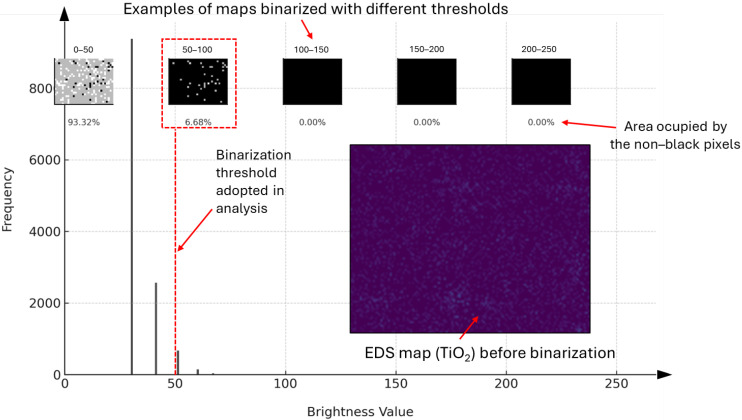
Histogram of a sample micro-area analyzed in the study, highlighting the binarization threshold used, with comparisons to other thresholds. (EDS parameters: magnitude—1000; HV—1.5 kV; tilt—0.0; detector—ADC1).

The analysis of the acquired EDS maps followed a systematic methodology. A monochromatic conversion was performed to facilitate subsequent processing for each EDS map, which was initial composed of RGB pixels. A static binarization threshold of 50 was consistently applied across all maps to enable a clear distinction between photocatalyst and non-photocatalyst regions (Figure 6). To assess the photocatalyst distribution on the mortar sample surfaces, a total of 42 EDS maps were generated for both the reference sample and those containing the maximum mineral powder content; each map covered contiguous micro-areas measuring 0.31 × 0.24 mm. The white pixel area in each binarized map was calculated, resulting in an aggregated analysis area of 2.17 × 1.44 mm (7 × 6 maps) per sample.

Microstructural imaging was conducted under a low vacuum (80 Pa) with an excitation voltage of 15 kV, employing a field emission scanning electron microscope (Nova NanoSEM 200, FEI, Hillsboro, OR, USA). The EDS analysis utilized an Octane Elect detector (EDAX) to capture the elemental distribution.

The grain size of mineral fillers and cement was determined using a HORIBA LA-300 laser grain size analyzer (Horiba Instruments Inc., Irvine, CA, USA) according to the methodology described in [34]. The density of the powder materials was determined using gas pycnometry. The chemical composition of the mineral fillers and cement was determined using the X-ray fluorescence (XRF) method using a spectrometer equipped with a lamp with a Rh anode (Axios Minerals, Almelo, The Netherlands). The percentage share of oxides was calculated using IQ+ software.

## 4. Results

### 4.1. Mechanical Properties

**Figure 7:**
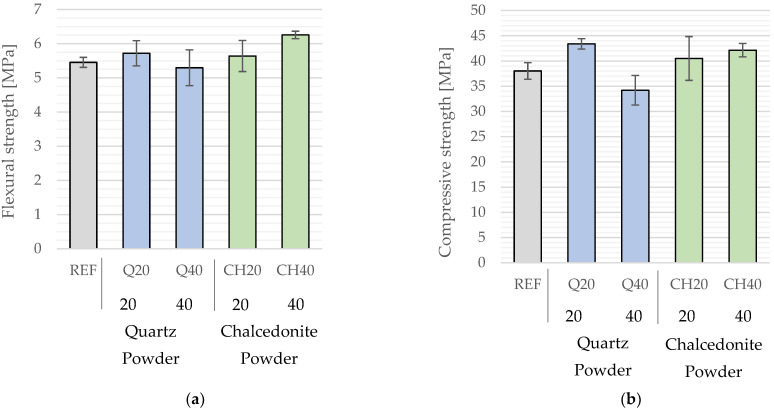
(**a**) Flexural strength. (**b**) Compressive strength of mortars as a function of mineral filler type and percentage substitution in binder mass. (Abbreviations: CH40—mortar with 40% chalcedonite filler by binder mass; Q40—mortar with 40% quartz filler by binder mass; REF—reference mortar with 0% filler).

The average flexural strength of the composites after a two-day curing period ranged from 5.30 MPa to 6.26 MPa (Figure 7a). Partial substitution of the cement with quartz filler showed no statistically significant effect on flexural strength, as indicated by the ANOVA (*p* = 0.41 > 0.05), thus supporting the null hypothesis. Conversely, replacing 40% of Portland cement with chalcedonite filler led to a statistically significant increase in flexural strength of approximately 15%.

The average compressive strength of the mortar samples ranged from 34.2 MPa to 43.4 MPa following a two-day curing period (Figure 7b). The highest compressive strength was observed in samples containing a 20% substitution of cement with quartz filler, while the lowest was found in those with a 40% substitution. The inclusion of quartz filler significantly influenced the compressive strength of the mortars, as confirmed by the ANOVA (*p* < 0.01). However, this effect was nonlinear: a 20% substitution of binder mass with quartz filler led to a compressive strength increase of approximately 14% compared to the control series, whereas raising the substitution to 40% resulted in a 10% decrease in strength. This reduction in performance at a higher filler content was likely due to the diminished workability of the fresh mortar, potentially creating macro-pores and defects within the cement matrix during binder hydration.

For mortars with chalcedonite filler, a linear trend of increased compressive strength was noted as the filler content rose. However, due to elevated standard deviations within these series, the statistical analysis indicated no significant effect of chalcedonite on compressive strength (*p* = 0.09 > 0.05, ANOVA).

### 4.2. Total Pore Content and Pore Network Characteristics

**Figure 8:**
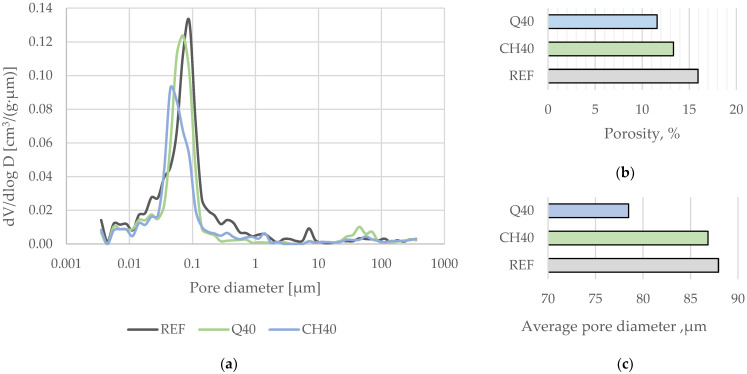
(**a**) Pore size distribution in the analyzed mortars. (**b**) Total porosity of the mortar samples. (**c**) Average pore size in the mortar samples. (Abbreviations: CH40—mortar with 40% chalcedonite filler by binder mass; Q40—mortar with 40% quartz filler by binder mass; REF—reference mortar with 0% filler).

The tested mortars predominantly contained mesopores within the 15–140 nm size range (Figure 8), accounting for approximately 73–81% of the total air voids. The average pore size across the samples ranged from 78 to 88 nm. Substituting Portland cement with quartz or chalcedonite filler reduced the overall porosity of the mortars, with this effect being more pronounced in mortars containing the coarser quartz filler. The quartz filler exhibited a lower water demand than the chalcedonite powder, a distinction observed during mortar preparation. Although all the mortar mixtures had similar flow characteristics, those with chalcedonite filler displayed a notably higher viscosity, evidenced by an extended flow time. Substituting 40% of the cement binder with quartz powder reduced the total porosity by approximately 4 percentage points, from 15.9% to 11.5%. In contrast, a 40% substitution with chalcedonic filler resulted in a more modest porosity reduction of less than 3 percentage points, from 15.9% to 13.3%.

### 4.3. Photocatalyst Distribution on the Sample’s Photoactive Surface

**Figure 9:**
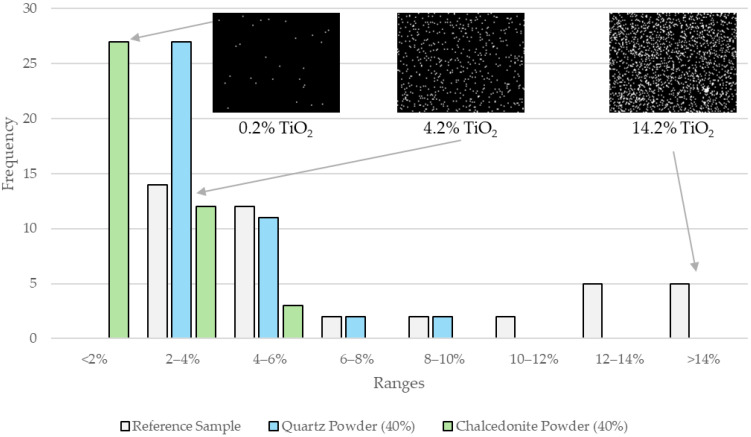
Histogram showing the distribution of the photocatalyst (number of micro-areas occupied by TiO_2_ within the specified ranges of the area) on the sample’s photoactive surface.

The mortars exhibited an irregular distribution of photocatalysts on their surfaces, as indicated by the non-uniform titanium signal distribution (Figure 9). After background subtraction, the titanium signal was detected over areas covering 0.2% to 14.4% of the analyzed sample surfaces.

The reference sample showed the strongest Ti signal, with the average area occupied by photocatalysts at 6.8%. In this sample, 10 of 42 micro-areas had a photocatalyst coverage exceeding 12% of the surface area. In addition, 16 out of 42 micro-areas showed a photocatalyst coverage above 6%. However, most of the analyzed areas (26 out of 42 micro-areas) had a TiO_2_ coverage within the 2–6% range.

In contrast, the mortars containing quartz and chalcedonite powders exhibited a much weaker Ti signal. No micro-areas with a Ti signal covering more than 10% of the surface were observed for mortars modified with quartz powder, although large TiO_2_ agglomerates, up to 150 μm in length, were present. In 27 out of 42 micro-areas, photocatalysts covered 2–4% of the photoactive surface, with an average coverage of 4.0%.

**Figure 10:**
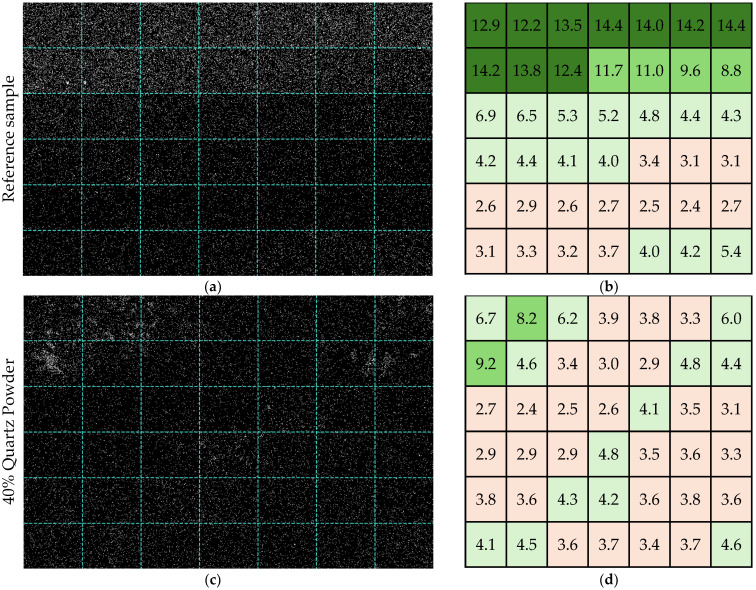

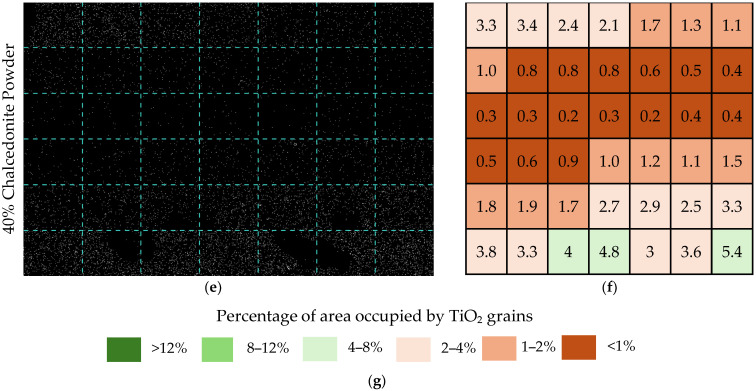
EDS mapping results (**a**,**c**,**e**) for titanium (Ti) after processing, showing the micro-areas of (**a**) the reference sample, (**c**) the sample with 40% quartz powder, and (**e**) the sample with 40% chalcedonite powder. (**b**,**d**,**f**) Tables showing the percentage of area occupied by TiO_2_ grains in each EDS map after processing. (**g**) Legend with color labels assigned to specific ranges of area values occupied by TiO_2_ (EDS parameters: magnitude—1000; HV—1.5 kV; tilt—0.0; detector—ADC1).

The weakest Ti signal was observed in the sample modified with 40% chalcedonite powder, where the average area occupied by photocatalysts was 1.8%. In this case, 27 of 42 micro-areas had a Ti signal coverage below 2.0%, with the lowest values around 0.2%. The highest observed value in the mortar modified with chalcedonite powder was 5.4%, and only three micro-areas showed a photocatalyst coverage above 4.0% (Figure 10).

### 4.4. Efficiency in Air Purification from NO_x_

**Figure 11:**
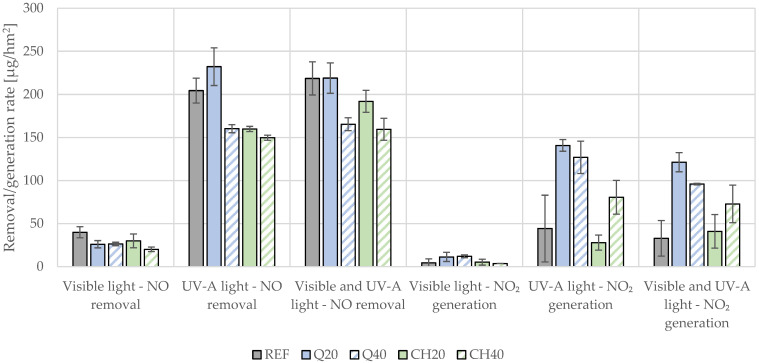
Photocatalytic removal rate of nitric oxides (NO) and generation rate of NO_2_ under investigated light conditions for mortars modified with mineral fillers as a function of mineral filler content (in terms of binder mass) and light source.

The photocatalytic efficiency for nitric oxide (NO) removal ranged from 20 to 40 µg/hm^2^ under visible light (VIS), 150 to 230 µg/hm^2^ under UV-a light, and 160 to 220 µg/hm^2^ when both light sources were active (Figure 11). A higher content of mineral fillers in the mortar composition corresponded with a reduction in photocatalytic efficiency. A 20% cement replacement with mineral fillers reduced the NO removal rate under all light sources for all modified photocatalytic mortars (except for Q20 in UV-a light, which was characterized by an average NO removal rate that was approximately 10% higher than the reference series but this was not a statistically significant effect due to the high coefficient of variation; ANOVA, *p* = 0.72 >> 0.05). Although the NO removal rate dropped by an average of 20–30%, the NO_2_ generation rate was significantly higher in the cases of the modified mortars. The Q20 series had the highest generation rate, which was more than threefold higher than the reference value (approx. 140 µg/hm^2^ for Q20 under UV-a irradiation compared to approx. 40 µg/hm^2^ for the reference series under the same light conditions).

**Figure 12:**
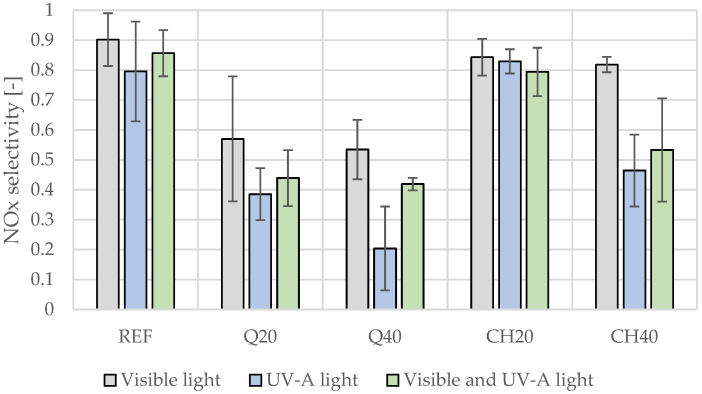
Selectivity of photocatalytic NO_x_ decomposition in relation to mineral filler content and light source.

The selectivity of nitrogen oxide (NO_x_) oxidation declined with increasing filler content in the binder (Figure 12). The reference samples, without fillers, exhibited the highest selectivity, ranging from 0.80 to 0.90, depending on the light source used. In contrast, all the binder series modified with mineral fillers demonstrated a reduction in selectivity. Specifically, the inclusion of quartz filler was associated with the greatest disruption in photocatalytic efficiency; in samples where 40% of the cement was replaced with quartz powder, the selectivity of the photocatalytic reaction declined to between 0.20 and 0.52, with the lowest values observed under UV-a light exposure.

While the rate of NO oxidation to NO_2_ per unit time remained comparable between the reference and filler-modified samples, the presence of fillers hindered further oxidation of NO_2_ to NO_3_^−^ ions. Among the tested samples, those with 40% replacement with chalcedonite powder exhibited the lowest NO removal rates. In these lower-selectivity samples, the reduced photocatalytic efficiency allowed a greater quantity of NO_2_ to desorb from the mortar surface under a gas flow and were detected by the analyzer. Notably, in the Q20 series under UV-a light, the NO_2_ emission rate was approximately threefold higher than that of the reference series. Given that NO_2_ poses a greater health risk than NO, the air-purification efficacy of mortars with a low oxidation selectivity is significantly limited.

## 5. Discussion

Photocatalytic cementitious composites are increasingly recognized for their potential to improve air quality in urban environments by effectively removing various gaseous pollutants, including nitrogen oxides (NO_x_) [35,36]. The final photocatalytic efficiency of a material is a superposition of multiple phenomena that affect the rate of chemical reactions initiated by electromagnetic radiation of a specific wavelength. The quality of the photocatalyst embedment on the surface of the composite [8], as well as other surface characteristics [37,38,39], contribute to the intensity of various photocatalytic reactions, increasing or decreasing the contact time between free radicals and pollutants. The composition of the composite can influence the distribution of nanometric photocatalytic modifiers throughout the volume of the composite, either facilitating or impeding the homogenous distribution of photocatalytic sites on the composite’s photoactive surface and influencing the effective area of the sample on which photocatalytic phenomena occur [16]. All of the aforementioned phenomena were impacted by the introduction of a mineral filler into the composite, diminishing its photocatalytic efficiency.

The primary distinction between the reference and modified mortar series was a significant reduction in the selectivity of NO_x_ photocatalytic reactions. This reduction implies that while the photocatalytic material was continuously exposed to electromagnetic radiation, the energy supplied or the effective contact time was insufficient to facilitate reactions beyond the initial oxidation of NO to NO_2_. Despite the irradiation conditions being consistent across all the series, this decline in photocatalytic efficiency points to two critical phenomena that may underlie the observed behavior. First, the agglomeration of TiO_2_ particles on the composite surface likely reduced the active surface area available for photocatalytic activity, impairing the material’s capacity to drive more advanced oxidation processes. Second, alterations in the porosity of the composite’s near-surface layer could have restricted the diffusion of reactant gases, such as NO and O_2_, to the active photocatalytic sites. These structural and surface-level changes hindered the material’s interaction with NO_x_ and disrupted the delicate balance required for optimal photocatalytic performance. Consequently, these findings underscore the importance of maintaining a well-dispersed TiO_2_ network and preserving the microstructural integrity of the composite to ensure sustained photocatalytic efficacy.

The investigated mineral fillers significantly altered the number of capillary pores (1.0–0.1 µm) near the composite surface, decreasing their volume from approximately 22.1% of the total pore network volume in the reference series to 13.0% in the Q40 series and 10.0% in the CH40 series. Given that the total pore volume varied between the series, it was found that in the case of the reference series, capillary pores constituted approx. 3.52% of total composite volume; for Q40, it was approx. 1.51%, and for CH40, it was approx. 1.34%.

Capillary pores play a pivotal role in facilitating fluid transport within the composite material, serving as conduits for various physical and chemical processes that occur within the near-surface zone of the exposed cement matrix [23,40,41,42]. These pores provide pathways for the reactants to diffuse toward the photocatalytic sites, enhancing the interaction between the active photocatalyst and the surrounding environment. However, when the volume of capillary pores is reduced, the available pathways for reactant diffusion are significantly limited, effectively confining the photoactive sites to those located directly on the composite surface. This reduction diminishes the overall photocatalytic efficiency and restricts the spatial extent of chemical reactions to the immediate surface layer.

Furthermore, the diminished porosity has profound implications for the photocatalytic reaction dynamics. Primary photocatalytic reaction products, such as NO_2_, are rapidly mobilized and removed by external airflow, a process that critically shortens the residence time of NO_2_ molecules in the vicinity of the TiO_2_ surface. This reduction in contact time severely limits the likelihood of secondary oxidation reactions, such as the conversion of NO_2_ into less harmful end products like nitrates (NO_3_^−^). Without sufficient interaction time between NO_2_ and the active sites of TiO_2_, the sequential reactions necessary for complete degradation of NO_x_ are impeded.

**Figure 13:**
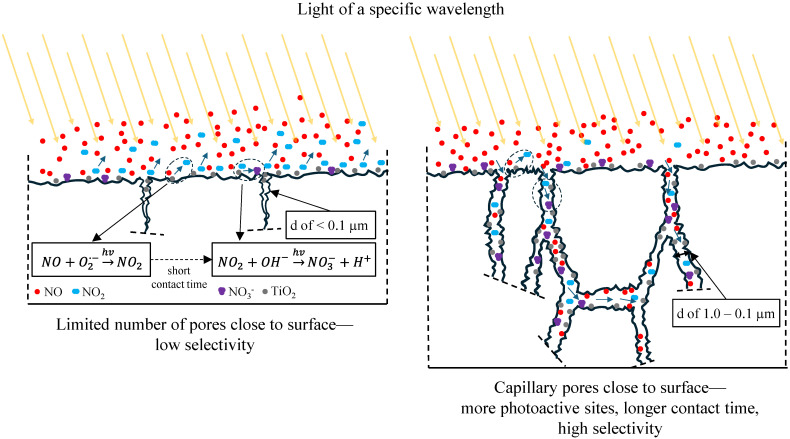
Schematic of the impact of pores close to the surface in the composite on the selectivity of photocatalytic reactions.

This phenomenon highlights the interplay between the composite’s microstructural characteristics and its functional performance. The loss of capillary pore volume impacts fluid transport and disrupts the delicate balance required for sustained photocatalytic activity. Optimizing the composite’s porosity during fabrication is crucial to mitigating these limitations, ensuring that adequate pathways are preserved to facilitate efficient reactant transport and prolong contact time with the photocatalyst (Figure 13).

A significant reduction in the TiO_2_ content near the surface of was detected in the cement with mineral fillers. The reference sample demonstrated the highest Ti signal intensity and photocatalyst coverage, with a significant portion of the micro-areas showing a higher TiO_2_ distribution across the surface. The quartz-modified sample showed no micro-areas with over 10% coverage, and the distribution was more consistent at lower coverage levels, averaging 4%. The chalcedonic-modified sample presented the weakest photocatalyst presence, with most micro-areas (64%) showing coverage below 2%. This gradient in Ti signal strength and surface coverage suggests that the composition of the matrix significantly affected the photocatalyst distribution, with the reference sample facilitating the highest photocatalyst retention. At the same time, the chalcedonite modifications resulted in the most limited photocatalyst exposure on the surface, which caused a reduction in the NO removal rate for the CH40 series during the air-purification tests compared to the reference mortar.

**Figure 14:**
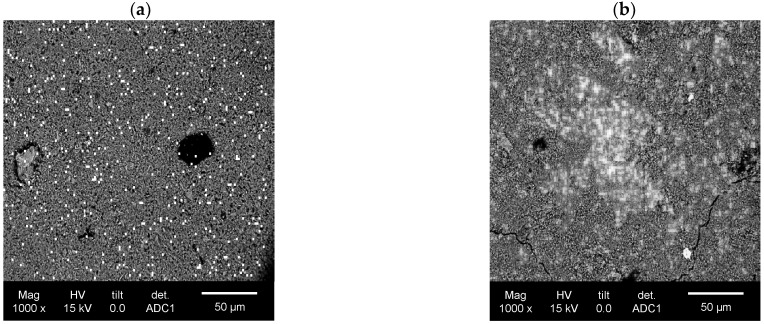
(**a**) Homogenous distribution of near-surface TiO_2_ grains of reference photoactive surface. (**b**) Agglomeration of near-surface TiO_2_ grains on the Q40 photoactive surface.

Additionally, the modifications contributed to the agglomeration of TiO_2_ grains in the near-surface layer of the composite, which, combined with a reduction in the porosity of the composite, further impeded its photocatalytic performance, reducing the specific surface on which photocatalytic reactions could occur (Figure 14).

Studies have demonstrated that a homogeneous distribution of nanometric TiO_2_ grains significantly enhances both the photocatalytic efficiency of photoactive surfaces by maximizing the interaction area between the photocatalyst and environmental pollutants, as well as optimizing the exposure to irradiation sources [8,21] and overall impact of nanomodification on the other properties of the composite [43]. In the current study, two primary mechanisms were identified as contributing to a non-uniform TiO_2_ distribution on the mortar surface. The first mechanism was linked to the rheological modifications induced by the inclusion of mineral fillers, which increased the viscosity of the composite. This viscosity increase is generally advantageous in self-compacting concrete (SCC) applications and other self-leveling cementitious composites, as it promotes uniformity by preventing segregation among mix constituents while preserving the mix’s fluidity. However, in the case of photocatalytic mortars, the increased filler content resulted in an undesirable reduction in TiO_2_ concentration in the surface layer, which is critical for photocatalytic performance.

**Figure 15:**
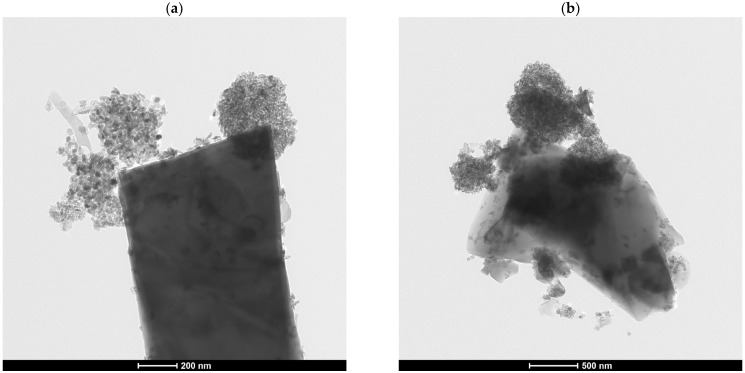
TEM micrographs of surface interactions between grains of the investigated mineral fillers ((**a**) quartz filler; (**b**) chalcedonite filler) and nanometric TiO_2_ grains used in the study; the agglomeration of TiO_2_ grains, with limited coverage over the filler grain external surface, was observed in both cases. The TEM analysis was performed on copper grids covered with a carbon film. (Microscope: TEM Tecnai TF 20 X-TWIN. Parameters: EDAX; voltage, 200 kV; the STEM images were collected using the HAADF detector).

The aforementioned reduction in TiO_2_ concentration was attributed to the space-occupying nature of the filler particles, which displaced the TiO_2_ particles, effectively diluting their concentration. This diminished surface concentration of TiO_2_ resulted in decreased photocatalytic activity due to a lower probability of contact between pollutants and active photocatalytic sites. Consequently, this decreased contact reduced both the overall photocatalytic efficiency and the selectivity of photoreactions, as the pollutants had reduced contact time with the TiO_2_. Furthermore, the issue could have been mitigated if the mineral fillers served as nano-carriers for TiO_2_ particles, facilitating their immobilization and uniform distribution throughout the cement matrix. Ideally, fillers with porous or structured surfaces could enable the partial adsorption of TiO_2_, allowing for a consistent dispersion of photocatalytic particles across the mortar’s exposed surface. However, in this study, the mineral fillers, quartz and chalcedonite, possess rigid, non-porous surfaces that lacked sufficient compatibility with TiO_2_. This incompatibility promoted the agglomeration of TiO_2_ into larger clusters, thereby decreasing the effective surface area available for light exposure and pollutant interaction (Figure 15).

## 6. Conclusions

The surface characteristics of photocatalytic cementitious composites play a crucial role in determining their overall air-purification efficiency, as these properties directly influence the exposure and accessibility of photoactive grains to environmental pollutants and external radiation [21,44,45,46]. In this study, comprehensive analyses were conducted to assess the impact of material composition and structural attributes on the distribution and effectiveness of TiO_2_ grains within the cement matrix of composites modified with mineral fillers. The findings underscore the importance of both the uniformity of TiO_2_ distribution and the interactions between fillers and photocatalytic agents in optimizing the composite’s photocatalytic response. Based on the results of this research, several key conclusions were drawn regarding the relationship between composite composition, surface properties, and photocatalytic performance:

- The partial replacement of binder mass with mineral fillers led to a measurable decrease in the air-purification efficiency of the investigated cementitious composites;
- The incorporation of both quartz and chalcedonite fillers significantly reduced the mortar porosity, affecting both the total pore volume and pore size distribution, with modified mortars exhibiting a marked decrease in capillary pore content;
- Alterations in the composite’s pore network characteristics impacted the photocatalytic reaction selectivity by reducing both the overall photoactive surface area and the interaction time between TiO_2_ particles and airborne pollutants, thereby diminishing the efficiency of the photocatalytic reactions,
- The inclusion of fine-grained mineral fillers resulted in a non-uniform distribution of TiO_2_ photocatalyst particles within the cement matrix, which further limited the extent of accessible photoactive sites on the composite’s surface,
- Due to the morphology of the filler grains (angular and characterized by a low roughness), they did not effectively act as carriers for the nanometric TiO_2_.

## Figures and Tables

**Table 1:** Mortar series investigated in this study.

Series ID	Mineral Filler	Water-to-Binder Ratio (w/b) [-]	Binder-to-Sand Ratio (b/s) [-]	Binder Composition [% b.m.]	
				Cement	Mineral Powder
REF	-	0.40	0.70	100	0
Q20	Quartz filler			80	20
Q40				60	40
CH20	Chalcedonite filler			80	20
CH40				60	40

**Table 2:** Characteristics of the cement binder used in the study.

Flexural Strength, MPa	Compressive Strength, MPa	Initial Setting Time, min	Final Setting Time, min	Specific Gravity, g/cm^3^	Specific Surface Area, cm^2^/g
4.46	44.4	190	270	3.09	3920

**Table 3:** Chemical composition of the binder and mineral fillers used in the study (LOI—loss on ignition).

Type	Chemical Composition [% of Mass]									Specific Gravity [g/cm^3^]
	CaO	SiO_2_	Al_2_O_3_	MgO	K_2_O	Na_2_O	Fe_2_O_3_	P_2_O_5_	LOI	
CEM I 42.5R	61.4	21.7	5.2	2.6	0.7	0.2	2.4	0.2	2.3	3.09
Chalcedonite powder	0.2	93.9	2.3	0.2	0.4	0.4	1.4	<0.1	1.5	2.63
Quartz powder	0.4	97.0	1.3	<0.1	0.2	0.1	0.2	<0.1	1.1	2.64

**Table 4:** The content of the crystalline phases (XRD), size of the crystallites (XRD), and specific surface area (BET) of the photocatalysts used in the study. XRD diffractograms of both photocatalysts are presented in Supplementary Material [Figure S1], providing a detailed comparison of their crystalline structures.

Photocatalyst	Size of Crystallites [nm]		Phase [%]		Specific Surface Area [m^2^/g]
	Rutile	Anatase	Rutile	Anatase	
TiO_2_ (A)	-	10	-	100	246.8 ± 2.9
TiO_2_ (B)	54	33	13	87	53.8 ± 0.2

**Table 5:** Properties and composition of fire-dried sand aggregate.

Property	Value
Granulation	0.1/0.5
Dust content	0.1%
Water absorption	0.1%
SiO_2_ content	99.6%
Fe_2_O_3_ content	0.01%
Al_2_O_3_ content	0.06%
Solubility in water	0.0%
Softening point	1506 °C
Sulfur content	<1%
Sand equivalent	99.06

## Data Availability

The data are included in the paper and are available on request.

## Supplementary Materials

The following supporting information can be downloaded at: https://www.mdpi.com/article/10.3390/ma17235775/s1, Figure S1. XRD diffractograms of: (a) K7000 photocatalyst, and (b) P25 photocatalyst.
