# Supplementary figures and images for: Effect of Cement Substitution with Mineral Fillers on NOx Air-Purification Efficiency and Photocatalytic Reaction Selectivity of Nano-TiO2-Modified Cementitious Composites
Source: Materials (Basel). 2024 Nov 25;17(23):5775. doi: 10.3390/ma17235775 (PMC11641952; doi:10.3390/ma17235775)

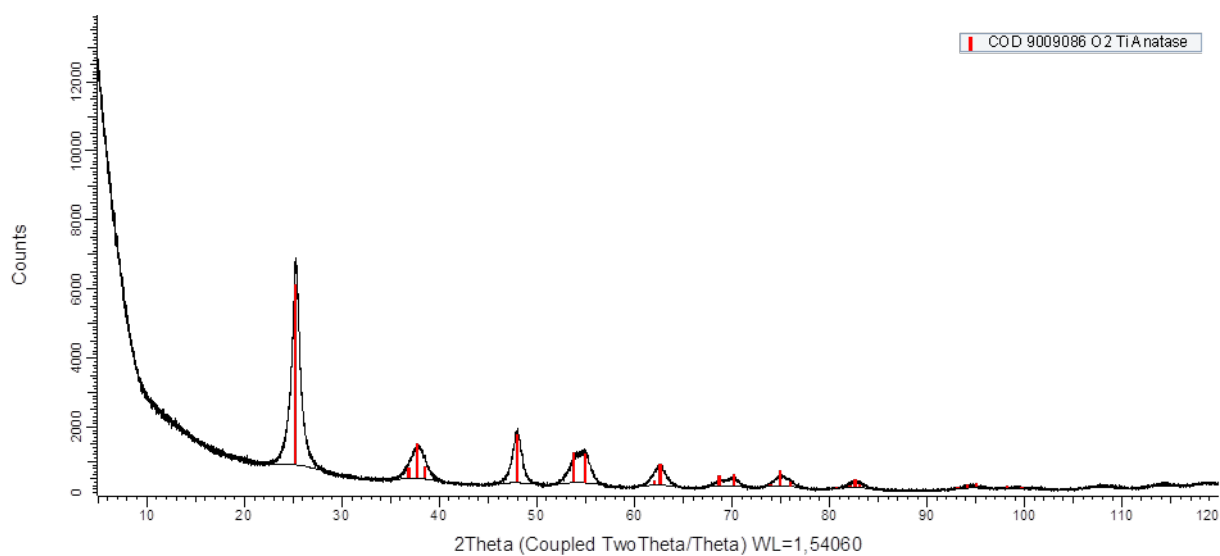

(a)

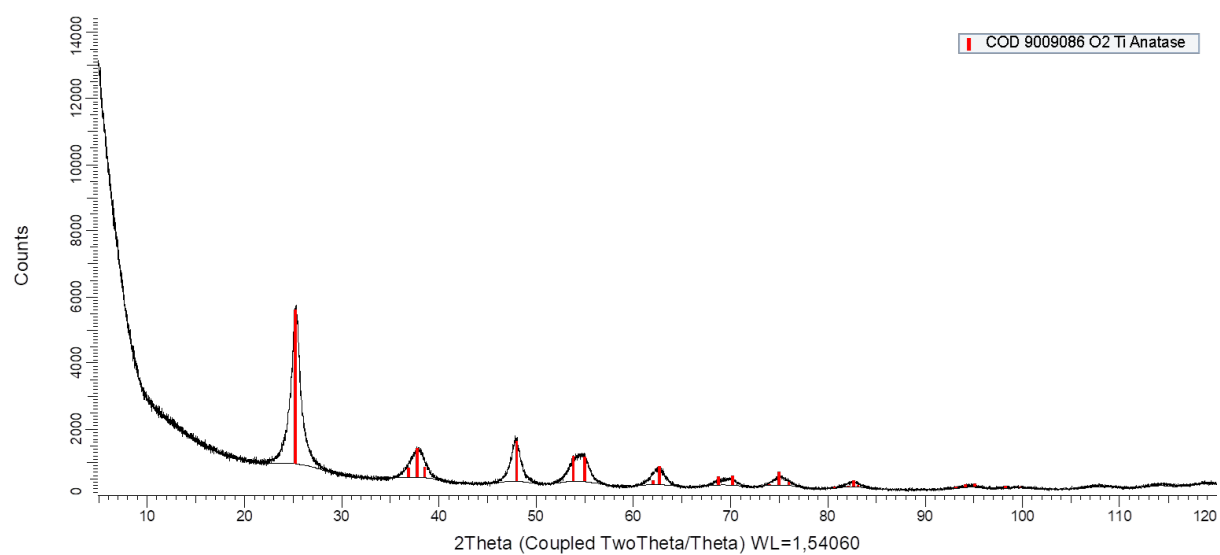

(b)

**Figure S1.** XRD diffractograms of: (a) K7000 photocatalyst, and (b) P25 photocatalyst.

Supplement: Supplementary file 1 [file materials-17-05775-s001.zip › materials-3328629-supplementary.pdf]
